# Expected Net Benefit of Vaccinating Rangeland Sheep against Bluetongue Virus Using a Modified-Live versus Killed Virus Vaccine

**DOI:** 10.3389/fvets.2017.00166

**Published:** 2017-10-11

**Authors:** Tristram R. Munsick, Dannele E. Peck, John P. Ritten, Randall Jones, Michelle Jones, Myrna M. Miller

**Affiliations:** ^1^Department of Agricultural & Applied Economics, University of Wyoming, Laramie, WY, United States; ^2^Agricultural Producer, Big Horn Basin, WY, United States; ^3^Department of Veterinary Sciences, University of Wyoming, Laramie, WY, United States

**Keywords:** domestic sheep, economics, intermountain west, Monte Carlo simulation, private cost, uncertainty, variability, Wyoming

## Abstract

Recurring outbreaks of bluetongue virus in domestic sheep of the US Intermountain West have prompted questions about the economic benefits and costs of vaccinating individual flocks against bluetongue (BT) disease. We estimate the cost of a BT outbreak on a representative rangeland sheep operation in the Big Horn Basin of the state of Wyoming using enterprise budgets and stochastic simulation. The latter accounts for variability in disease severity and lamb price, as well as uncertainty about when an outbreak will occur. We then estimate the cost of purchasing and administering a BT vaccine. Finally, we calculate expected annual net benefit of vaccinating under various outbreak intervals. Expected annual net benefit is calculated for both a killed virus (KV) vaccine and modified-live virus vaccine, using an observed price of $0.32 per dose for modified-live and an estimated price of $1.20 per dose for KV. The modified-live vaccine’s expected annual net benefit has a 100% chance of being positive for an outbreak interval of 5, 10, or 20 years, and a 77% chance of being positive for a 50-year interval. The KV vaccine’s expected annual net benefit has a 97% chance of being positive for a 5-year outbreak interval, and a 42% chance of being positive for a 10-year interval. A KV vaccine is, therefore, unlikely to be economically attractive to producers in areas exposed less frequently to BT disease. A modified-live vaccine, however, requires rigorous authorization before legal use can occur in Wyoming. To date, no company has requested to manufacture a modified-live vaccine for commercial use in Wyoming. The KV vaccine poses less risk to sheep reproduction and less risk of unintentional spread, both of which facilitate approval for commercial production. Yet, our results show an economically consequential tradeoff between a KV vaccine’s relative safety and higher cost. Unless the purchase price is reduced below our assumed $1.20 per dose, producer adoption of a KV vaccine for BT is likely to be low in the study area. This tradeoff between cost and safety should be considered when policymakers regulate commercial use of the two vaccine types.

## Introduction

Bluetongue (BT) is an insect-borne, hemorrhagic, viral disease that affects domestic sheep and other ruminants throughout much of the world, including the US (1). Clinical signs of bluetongue virus (BTV) infection in sheep (*Ovis aries*) can range from mild to severe or fatal; they include widespread edema, internal hemorrhaging, nasal discharge, weight loss, and oral ulcerations. The risk of BT disease can be reduced through vector control (e.g., insecticide application) or vaccination of susceptible flocks. Vector control has been helpful in Europe and Asia (2). However, vaccination has been the most effective method for preventing and controlling BT in Europe (3).

**Figure 1:**
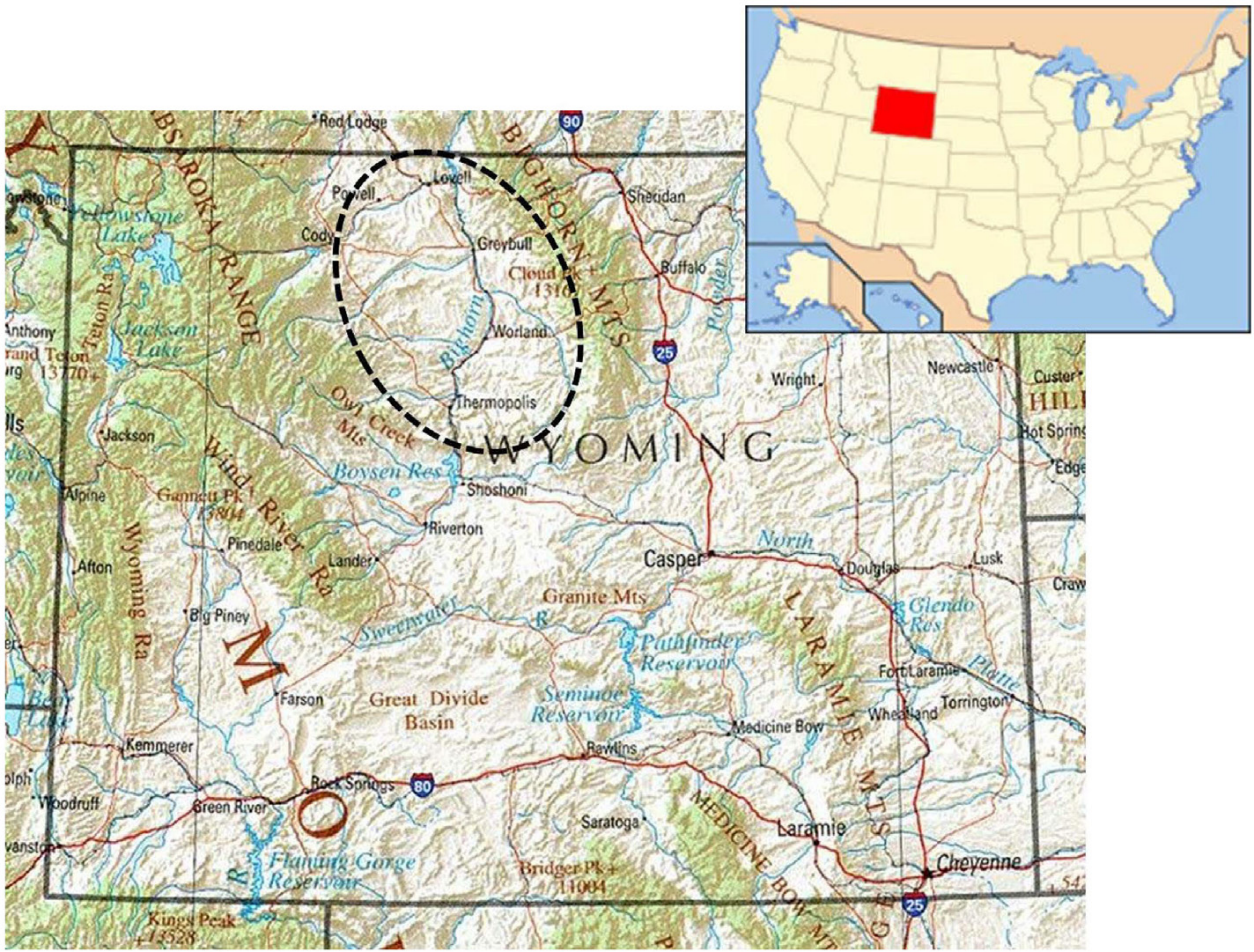
Map of the study area, Big Horn Basin, in the state of Wyoming, USA. Images courtesy of the University of Texas Libraries, The University of Texas at Austin (below) and https://en.wikipedia.org/wiki/Outline_of_Wyoming#/media/File:Map_of_USA_WY.svg (top).

Virus serotypes, vaccine regulations, and day-to-day management practices on rangeland sheep operations in the US Intermountain West are sufficiently different from those in Europe that they may alter the economic effectiveness of vaccination. Costs and benefits of vaccination against BT in the US Intermountain West have not previously been estimated. This is due, in part, to a lack of research on the economic consequences of BT outbreaks in this region. We, therefore, estimate the costs of a BT outbreak in a representative rangeland sheep flock in the state of Wyoming, as well as the costs and expected benefits of vaccinating against BTV. Wyoming is an important sheep-producing state, currently ranking fourth in the US for lamb output, and accounting for 6.7% of the total US sheep inventory (4). The Big Horn Basin of north-central Wyoming (roughly 8,000 km^2^; Figure 1), in particular, experienced a severe BT outbreak in 2007, which provides a case-study on which to base many of our model parameters and assumptions.

We analyze the economic costs and benefits of two different vaccine types: killed virus (KV) and modified-live virus (MLV). A sheep producer in Wyoming can currently obtain a KV vaccine legally for commercial use, but it involves custom-manufacturing and is, therefore, not readily available and thought to be more expensive. An MLV vaccine, in contrast, cannot be legally obtained for use in Wyoming—due to vaccine safety concerns—yet, is thought to be less expensive and hence more likely to be adopted. These tradeoffs between vaccine safety and vaccine cost (thus adoption) have piqued the interest of animal health policymakers in Wyoming. Unfortunately, the epidemiological data necessary for quantifying the economic value of vaccine safety are not currently available in our study area. But we lay the groundwork for future research and policy debate by determining if either vaccine generates enough benefits to outweigh the private costs of purchasing and administering it. If both vaccines are too costly to justify private investment by sheep producers, then future research and policy debate may be unnecessary. But if an MLV vaccine proves economically attractive for producers, while a KV vaccine does not because of its higher cost, then further research and policy debate may be justified.

### BTV and Disease

Bluetongue is a non-contagious, insect-borne viral disease that afflicts sheep, cattle, and other ruminant species (5). BTV is transmitted between susceptible animals by particular biting-midge species of the genus *Culicoides* (*C. sonorensis* in Wyoming). The disease’s distribution depends on the range of the relevant midge species, but has historically circumnavigated the globe in temperate and semi-arid climates, between approximately 40° North and 35° South (6). Although BTV has a wide global distribution, symptomatic occurrence of BT disease is most common at the northern and southern boundaries of the virus’ range. Virulence is significantly lower in areas where BTV is endemic (i.e., chronically prevalent). This may be due to increased population immunity and co-evolution of the virus and its susceptible hosts (7).

Bluetongue virus, the etiological agent of BT disease, is a member of the *Orbivirus* genus within the *Reovirideae* family. Evolution of BTV is driven by genetic drift, shift, and intragenic recombination (8). Over time, this has led to the establishment of at least 27 known viral serotypes worldwide. In the US, five serotypes were historically identified (BTV 2, 10, 11, 13, and 17), and 10 additional BTV serotypes were recently identified in the southeastern US (7). Viral serotypes vary from each other in characteristics such as virulence and transmission potential (9–11). This may help explain the highly variable morbidity and mortality rates experienced during outbreaks of different viral strains around the globe.

Clinical signs of BT vary in severity depending on the strain of virus and host species affected. Sheep, deer, and antelope (wild or domestic) are relatively more susceptible and affected, whereas cattle and goats are less affected (12–15). In severe cases, the infected ruminant’s tongue becomes swollen and discolored and may protrude from the mouth, hence the name “bluetongue.” Gross legions on the heart, liver, spleen, kidneys, lungs, and gastrointestinal tract may have extensive hemorrhaging. The course of the disease ranges from 2–15 days, with the majority of symptoms usually appearing within 7 days of infection (16). In mild cases, recovery is swift with costs consisting primarily of weight loss and supportive care. In more severe cases, often in previously unexposed (i.e., naïve) populations, recovery may be prolonged and generate much higher losses. Mortality rates under typical Intermountain West field conditions vary between 4 and 20% of the total exposed population. Death typically occurs 1–8 days after the appearance of symptoms (16).

### Economic Costs of BT Outbreaks

Most recent economic studies of BT disease have been conducted in Europe, where several outbreaks of BTV-8 (BTV, serotype 8) have had severe effects on the sheep and cattle industries (17). Most notably, epidemics in the Netherlands during 2006 and 2007 caused large economic losses to sheep and cattle producers. The 2006 outbreak affected 460 farms in the Netherlands, with additional outbreaks occurring in neighboring Belgium, France, Germany, and Luxemburg. The epidemic of 2007 affected more than 6,000 farms in the Netherlands alone. Economic losses totaled 32 million Euros in 2006 and 175 million Euros in 2007 (17). Although the majority of losses occurred within the cattle industry, the sheep industry was also impacted. Within the sheep sector, breeding farms suffered the greatest losses (58% of total sheep morbidity and mortality losses for 2006; 72% of losses for 2007) (17). Velthuis et al. (3) show that vaccination of all adult sheep and cattle is the best strategy for BT disease control and prevention in Europe, based on benefit–cost ratios.

Although studies estimating the economic consequences of BT outbreaks in Europe offer useful insights, they focus on virus serotypes that do not exist in the US Intermountain West (9). Furthermore, the regulatory and physical environment, scale, and management practices of European sheep operations differ enough from the Intermountain West’s extensively managed rangeland sheep flocks to necessitate a separate study.

### Recent BT Outbreaks in the US Intermountain West

In 2007, a regional outbreak of BTV-17 (BTV, serotype 17) was first identified in several sheep flocks in the southeastern region of the state of Montana. The disease was subsequently reported in northern regions of the state of Wyoming; first in pronghorn, white-tailed deer, and mule deer in early fall of 2007, and finally in domestic sheep in the Big Horn Basin of northern Wyoming in late fall of 2007 (16). Quarantines were placed on sheep flocks in 17 Montana counties and 3 Wyoming ranches, preventing any off-farm movement.

Bluetongue virus-17 outbreaks had previously occurred in other parts of Wyoming and Montana; however, ranchers and veterinarians reported that Big Horn Basin flocks had not been previously exposed (16). This may be due, in part, to the region’s surrounding mountain ranges, which act as a natural barrier to foreign vector populations (16). Outbreaks occur more regularly in other regions of Wyoming that are less geographically protected. In such regions, previously exposed sheep populations seem to experience less severe symptoms than those sheep affected during the Big Horn Basin epidemic. These anecdotal observations provide grounds for further investigation of some veterinarians’ hypothesis that sheep populations can build immunity over time if exposed to the virus regularly (16).

The BTV-17 outbreak in Big Horn Basin during 2007 had severe consequences on regional sheep operations. One flock suffered 36% morbidity (500 out of 1,404 sheep) and 20% flock mortality (275 out of 1,404 sheep) (Personal communication with Ranch A operator in 2015). A neighboring ranch was also affected, but fared slightly better: 14% morbidity (233 out of 1,679 sheep) and 0.2% flock mortality (7 out of 1,679 sheep) (16). Recovery from an epidemic of this magnitude can be challenging, given that western US livestock ranches typically have net returns averaging 2–4% per year (18). This thin profit margin can quickly disappear during an unexpected disease epidemic.

### BT Prevention Using Vaccines

Vaccination has been used effectively in some regions of the US to control BT in livestock. But because of the region-specific diversity of BT strains, there is limited demand for a vaccine against any one strain. Limited demand has prevented broad-scale commercialization of BT vaccines. Exceptions include a live-attenuated (or modified-live) vaccine against BTV-10, which is approved for use throughout the US, and live-attenuated vaccines against BTV-10, 11, and 17 that are approved for use only in California (19).

Another reason for limited availability and approval of BT vaccines in the US is safety concerns, which differ for the two vaccine types we analyze here. The first vaccine type, live-attenuated or MLV, are relatively cheap to produce and are routinely used in domestic sheep flocks throughout Israel, South Africa, and some US states (limited to certain strains) (20). But use of MLV vaccines is prohibited in some US states because of their potential to revert to virulent type, as well as their ability to infect and be transmitted by insect vectors, thereafter circulating as a field strain. MLV viruses also have the ability to reassort gene segments with field viruses and create novel progeny (7). Finally, if given during pregnancy, MLV vaccines may cause unintended abortions, deformities, and other pregnancy complications (7).

The second type of vaccine is an inactivated autogenous, or KV vaccine. These are typically more expensive to produce than MLV vaccines and require a follow-up dose to attain a protective level of antibodies (19). However, they do not suffer the safety risks associated with MLV vaccines. Specifically, KV vaccines do not revert to virulence, do not reassort genes with field viruses, and do not cause reproductive damage to pregnant females (7).

Speiser et al. (21) tested the ability of two different custom-made BTV-17 vaccines—KV and MLV—to trigger a humoral response in ewes from seven commercial sheep operations in Wyoming. Both vaccines induced protective levels of antibodies, which lasted for at least 1 year and provided passive immunity for lambs. In light of the equal effectiveness of both vaccine types, the next step is to evaluate and compare the vaccines’ economic performance.

Our study evaluates the economic costs and expected benefits of vaccinating Wyoming domestic sheep flocks against BTV-17, using either a KV or MLV vaccine. Our results can help inform discussions between sheep producers, vaccine manufacturers, the State Veterinarian, and Livestock Board about potential approval and commercial sale of a BTV-17 vaccine for Wyoming. Currently, Wyoming producers can custom-order a KV vaccine manufactured from a recent isolate taken from an outbreak occurring in their region. An MLV vaccine for BTV-17 is not currently available for producer use outside of California, but the potential exists to legalize its importation into western sheep-producing states such as Wyoming (Personal communication with Dr. Jim Logan, Wyoming State Veterinarian in 2016). Before engaging in a debate about MLV vaccine legalization, animal health policymakers need to know if either vaccine would generate positive economic net benefits for producers.

## Materials and Methods

### Expected Profit Maximization

The theoretical framework for this study is expected profit maximization. We assume a sheep producer’s expected profit depends on their decision to vaccinate (or not vaccinate) their flock against BT. When combined with two possible states of nature (BT affects their flock, or it does not), there are four possible outcomes: (1) vaccinate and sheep contract BT anyway; (2) vaccinate and sheep do not contract BT; (3) do not vaccinate and sheep contract BT; (4) do not vaccinate and sheep do not contract BT. The producer organizes these four possible outcomes into two different expected profit functions: one for the decision to vaccinate, and one for the decision not to vaccinate. They then compare the two expected profits to decide whether or not to vaccinate.

The first step in identifying the expected profit maximizing decision is to determine the probability of occurrence for each of the four possible outcomes. Our chosen probabilities are based on historical data of the disease (16) and first-hand producer knowledge for the Intermountain West (Personal communication with Ranch A operator in 2016). To represent differences in disease prevalence throughout Wyoming, we calculated the expected net benefit associated with outbreaks occurring every 5, 10, 20, or 50 years.

### Rangeland Sheep Production Budgets

The next step of our analysis is updating an existing sheep enterprise budget, for a representative operation with 640 breeding ewes (1,404 sheep in total) (22), to US$2014 prices using indices of prices paid by Wyoming farmers and ranchers from 2010 to 2014 (23). Prices assumed in our analysis are reported in Table S1 in Supplementary Material. The updated budget provides an outline of management activities, costs, and revenues for a 1-year production calendar. It provides baseline estimates of profit, which we later adjust to reflect the costs of vaccinating sheep against BT, and the cost of a BT outbreak itself. A summary of the baseline budget is available in Table S2 in Supplementary Material. Munsick (24) provides a more detailed version of the baseline budget.

### BT Outbreak Costs

To estimate the cost of a BT outbreak, we reconstruct the series of events experienced by an anonymous producer during the 2007 BT outbreak in the Big Horn Basin of Wyoming. The dates they report imply a particular subset of production activities being disrupted. If the outbreak’s timing had differed substantially from this, an alternative subset of activities would be disrupted, resulting in different outbreak costs. The anonymous producer’s outbreak unfolded as follows. BT symptoms were detected September 1st and ended October 15th, encompassing 45 days of symptomatic disease within the flock. An on-site flock quarantine was imposed throughout the duration of the outbreak. The end of vector season occurred on the first hard freeze (29°F or below) on October 15th (23). An additional asymptomatic quarantine period of 14 days commenced on October 16th and extended to October 30th. This lengthened the total BT outbreak quarantine period to 59 days (Personal communication with Ranch A operator in 2015).

Tangible costs that a producer incurs during an outbreak include death loss, supportive care, pharmaceuticals, loss of condition, labor, and veterinarian fees. Intangible costs, such as stress and other emotional impacts, are not quantified here but are no less impactful. To calculate lamb death loss, we multiply the number of lambs lost to BT by a 25-year mean market value, $130/cwt, which we derive from a distribution of real historical lamb prices (1990–2014) from the Centennial Livestock Auction near Fort Collins, CO, USA (25). We assume that our representative operation markets 90-pound feeder lambs.

Ewe death loss is calculated by multiplying the number of ewes lost to BT by the economic value of an average-aged ewe in the flock. Based on conversations with regional producers (Personal communication with Peter John Camino, former President Wyoming Woolgrowers Association and with Ranch A operator in 2015), our representative flock’s replacement ewes are developed from within the operation. Therefore, the loss of an adult ewe due to BT is considered a capital loss. Specifically, it is equal to the cost of developing an identical replacement minus the cull value. This capital loss is then added to the discounted cull value lost to determine overall economic value lost when a ewe dies from BT. Details of the cost to develop a replacement ewe can be found in Munsick [(24), p. 16]. We use a similar method to calculate costs associated with ram death loss from BT [(24), p. 17]. Throughout our analysis, we use a 7% rate to discount the operation’s future costs and benefits to the present. This rate is commonly used for agricultural investments; it accounts for a 4% real rate of return on investment plus a 3% risk premium (18).

Another factor in the cost of a BT outbreak is providing supportive care to infected sheep. Substantial swelling in the face and throat of infected sheep requires a producer to provide a source of nourishment other than forage or hay. Ranch A accomplished this by mixing creep feed with water and administering the mixture directly to infected animals *via* feeding tube. Infected sheep that eventually recover begin to show signs of improvement after 7 days of creep-feeding. Those that eventually die do so after 10 days of creep-feeding (Personal communication with Ranch A operator in 2015). Infected lambs consume 2 lbs of creep feed (before adding water) per day while ewes and rams consume 3 lbs per day.

Pharmaceuticals needed during an outbreak are also included in supportive care costs. Direct vector control, for example, is beneficial when used to supplement vaccination in preventing further BT infections once an outbreak is detected [(2); Personal communication with Dr. Jim Logan, Wyoming State Veterinarian in 2016]. Permethrin is an affordable, widely available insecticide approved for direct use on livestock, including sheep. It is helpful in the control of the BT vector, *Culicoides* spp. (2). However, application is labor intensive [(2); Personal communication with Ranch A operator in 2015], and its effectiveness as a sole control measure (which we do not quantify in this analysis) is incomplete (26). We assume the treatment is repeated every 2 weeks throughout the course of the outbreak, totaling three treatments from September 1st to October 15th.

A significant portion of BT mortality is caused by secondary respiratory infections (16). Nuflor is an example of a synthetic, broad-spectrum antibiotic that aids in prevention and treatment of bacterial pathogens (e.g., pneumonia) that commonly occur during a BT outbreak (Personal communication with Dr. Matt Cherni, practicing large animal veterinarian in 2015). Nuflor label instructions call for a two-dose treatment, the second being administered 48 h after the first. Dexamethasone is used as an anti-inflammatory and may be administered simultaneously with Nuflor to the entire flock. The drug is relatively affordable and is effective in treating inflammatory symptoms common for BT, such as fever, pain, and swelling.

Due to a lack of data, we assume no loss in ewes’ reproductive efficiency or condition during a BT outbreak. However, we do account for a loss of condition in market lambs during the year of the outbreak. We assume a 10% live-weight loss (i.e., 90 lb lamb × (1 − 0.10) = 81 lb live weight, or a 9 lb loss per lamb) in surviving infected lambs which are marketed soon after recovery (Personal communication with Ranch A operator in 2015).

Labor costs include additional hired labor required to manage a BT outbreak. Many operations will fulfill additional labor requirements with longer hours for themselves and their families, but we assume additional owner labor is unavailable and, therefore, hired labor must be increased. The amount of additional labor necessary to cope with an outbreak is estimated based on Ranch A operator’s experience in 2007.

A BT outbreak generally does not involve extensive veterinarian resources. However, some visits from State and Federal officials may be necessary. The use of these public resources represents a social cost (as opposed to private cost) of the disease, and should not be overlooked. Similarly, research funded with public dollars to better understand BT represents an additional social cost (and social benefit). Our study focuses on private costs to sheep producers and, therefore, does not attempt to estimate the public cost of State or Federal veterinarians’ visits, BT research, or other public resources used during an outbreak.

### BT Vaccination Costs

The cost of purchasing and administering a BT vaccine is based on pharmaceutical companies’ recommendation that producers annually vaccinate their entire flock. To improve efficiency and decrease vaccination labor costs, we assume that a producer vaccinates their entire flock at the same time as deworming, on June 1st. This also allows for passive immunity to begin to wane in the newborn lamb crop (Expert opinion of co-author, Miller). Since the entire flock is already being handled and run through the chute for deworming, we assume it takes only 10 additional seconds to vaccinate for BT. The follow-up dose, required only if using the KV vaccine, can be given at any point after a minimum of 3 weeks has passed since the first dose. However, for the vaccine to produce desired levels of antibodies, the second dose must be given at least 7 days prior to flock exposure to the disease. For many producers, this amounts to vaccinating their flock twice before moving to summer range, or possibly administering the follow-up dose at some point during summer. If producers decide to vaccinate their flock separately from any existing handling sessions, the cost of labor will increase beyond what is reported in our analysis. Total vaccination costs are calculated by summing the cost of purchasing the vaccine and the cost of labor needed to administer it during existing handling sessions.

#### MLV Vaccination Cost

An MLV vaccine contains whole viruses that are able to grow and multiply within a host body. They stimulate the host immune system to create antibodies, yet do not typically cause actual disease in the host. Because the vaccine contains live active viruses, only one annual injection is needed (27).

A major side-effect of the MLV BT vaccine is the potential to cause abortions in pregnant ewes or malformations in their lambs. We do not include risk of abortions in our cost estimate, because we assume that producers administer the vaccine properly, i.e., not during pregnancy. There is also a risk that the modified virus will revert to virulent type, causing the vaccinated animal to become sick, or to infect and be transmitted further by the vector midges (28). This “escape” risk represents an external or social cost and is not borne by producers directly. Therefore, it is outside the scope of this analysis, which focuses solely on producer’s private costs. These two risks limit the use of an MLV vaccine to between March 31 and June 1 (i.e., after lambing and before the height of vector season).

The only available MLV vaccine for BTV-17 is approved for use strictly in California. For use in Wyoming, the producing company would need to seek USDA approval for distribution and use in other states, and then solicit a formal request from the State Veterinarian. To do this they must provide adequate documentation of efficacy, product safety, and USDA licensure (Personal communication with Dr. Jim Logan, Wyoming State Veterinarian in 2015). We assume a retail purchase price for such an MLV vaccine of $0.32 per dose, based on the price advertised for a similar vaccine on the California Wool Growers Association’s website (http://cawoolgrowers.org/vaccines/bluetongue.html).

#### KV Vaccination Cost

An autogenous or KV vaccine is produced using virus strains isolated from infected tissue samples using a cell culture system (29). As a USDA-licensed, restricted-use product, KV vaccines against BT are available for use only under veterinarian supervision. However, they involve no seasonal restrictions on its use, no risk of vector transmission, and no risk of abortion if used in pregnant ewes. The vaccine can be made as a monovalent (single antigen), bivalent (double antigen), or trivalent (triple antigen) vaccine. However, our analysis focuses on a monovalent vaccine for BTV-17.

Production of a KV vaccine takes approximately 12 weeks. Therefore, production and field-deployment of this type of vaccine might be infeasible if begun after the onset of an outbreak. However, virus isolates taken from a specific site may be used for up to 15 months from the date of isolation from tissue (30). An additional 24 months of use may be granted if vaccine efficacy has been shown and a viable threat of disease still exists. The use of a KV vaccine is also restricted to the BTV isolate’s source flock. However, permission for use in other flocks may be granted by the State Veterinarian (30). Retail pricing for a KV vaccine is assumed to be approximately $1.20/dose, which includes a 20% markup by a private veterinarian (Personal communication with Newport Laboratories, Worthington, MN, USA, in 2012).

### BT Vaccination Benefits and Net Benefits

Recent studies have found the efficacy of both MLV and KV vaccines under controlled conditions to range from 84 to 100% (21). We assume a conservative 84% effectiveness for both vaccines. Vaccine effectiveness is modeled by reducing the number of infected sheep by 84%, which propagates through the enterprise budget by reducing the number of sheep experiencing morbidity or mortality and the total hours of supportive care required.

The next step in our analysis is to calculate expected annual net benefit of vaccinating. We calculate this for both the MLV and KV vaccine, and for outbreak intervals of 5, 10, 20, and 50 years. To calculate expected annual net benefit, we first determine the expected benefit of vaccinating in a given year, accounting for time value of money and uncertainty in outbreak timing. Even if a producer knows the outbreak interval for their area (e.g., 10 years), they cannot predict the exact year in which an outbreak will occur. Therefore, we calculate the present value (PV) of benefit for each possible year in which the outbreak could occur (e.g., 1–50). We then annualize each of these PVs. Next, we multiply each annualized benefit by the probability of an outbreak occurring in a given year (e.g., 0.02). This generates a weighted annualized benefit for each year. Finally, we sum the weighted annualized benefits for each year to obtain an average or expected annual benefit over the given outbreak interval. This approach accounts for time value of money as well as uncertainty about an outbreak’s timing within a given interval. From this expected annual benefit, we then subtract the annual cost of vaccination to obtain expected annual net benefit of vaccination. This four-step calculation is summarized in Eq. 1:

(1) $$\text{Expected Annual Net Benefit}=\sum_{t=0}^{N-1}\left[\frac{\text{Benefit}}{{\left(1+i\right)}^{t}}*\frac{i}{1-{\left(1+i\right)}^{-N}}*\frac{1}{N}\right]-\text{Annual Cost},$$

where *N* is the outbreak interval (e.g., *N* = 5, 10, 20, or 50); *t* is the year in which an outbreak occurs (e.g., *t* = 0 indicates it occurs in the current year); *Benefit* is the undiscounted benefit of having a vaccinated flock when an outbreak does occur; *i* is the discount rate; and *Annual Cost* is the real cost incurred per year to vaccinate a flock against BT disease. In summary, the first term inside the brackets discounts the benefit of vaccinating based on the year in which the outbreak occurs. This adjusts for a producer’s time value of money (i.e., $1 of benefit received today tends to be valued more highly than $1 of benefit received in a future year). Next, the middle term inside the brackets annualizes the PV of benefit, spreading it evenly across the entire outbreak interval after accounting for the discount rate. The third and final term inside the brackets weights the annualized benefit by its probability of occurring (i.e., by the probability of an outbreak occurring in year, *t*). This three-step process is repeated for every *t* (i.e., for every possible year in which the outbreak could occur, given a particular outbreak interval). The resulting set of weighted annualized benefits is then summed to give the expected annual benefit. Finally, annual cost of vaccinating a flock is subtracted from expected annual benefit, resulting in expected annual net benefit of vaccination.

### Incorporating Variability: @Risk Simulation

For the final step of our analysis, we use the software program @Risk to conduct a Monte Carlo simulation, which allows three parameters in our model to vary (26): lamb price, morbidity rate, and mortality rate. Monte Carlo simulation is a technique that randomly draws a set of parameter values (where each set represents an “iteration”) from a probability distribution (29), and then uses those values in all calculations involving the three parameters. Each simulation involves running 50,000 iterations.

By simulating variability in lamb price, morbidity rate, and mortality rate, we are able to account for the inherent variability of outbreaks occurring in different years and at different geographic locations. Producers are generally well-aware of changing market and disease conditions and, therefore, desire information about vaccine performance during worst-case and best-case scenarios, not just on average. The distributional information generated through Monte Carlo simulation helps producers identify strategies that are robust to a range of possible outcomes.

**Figure 2:**
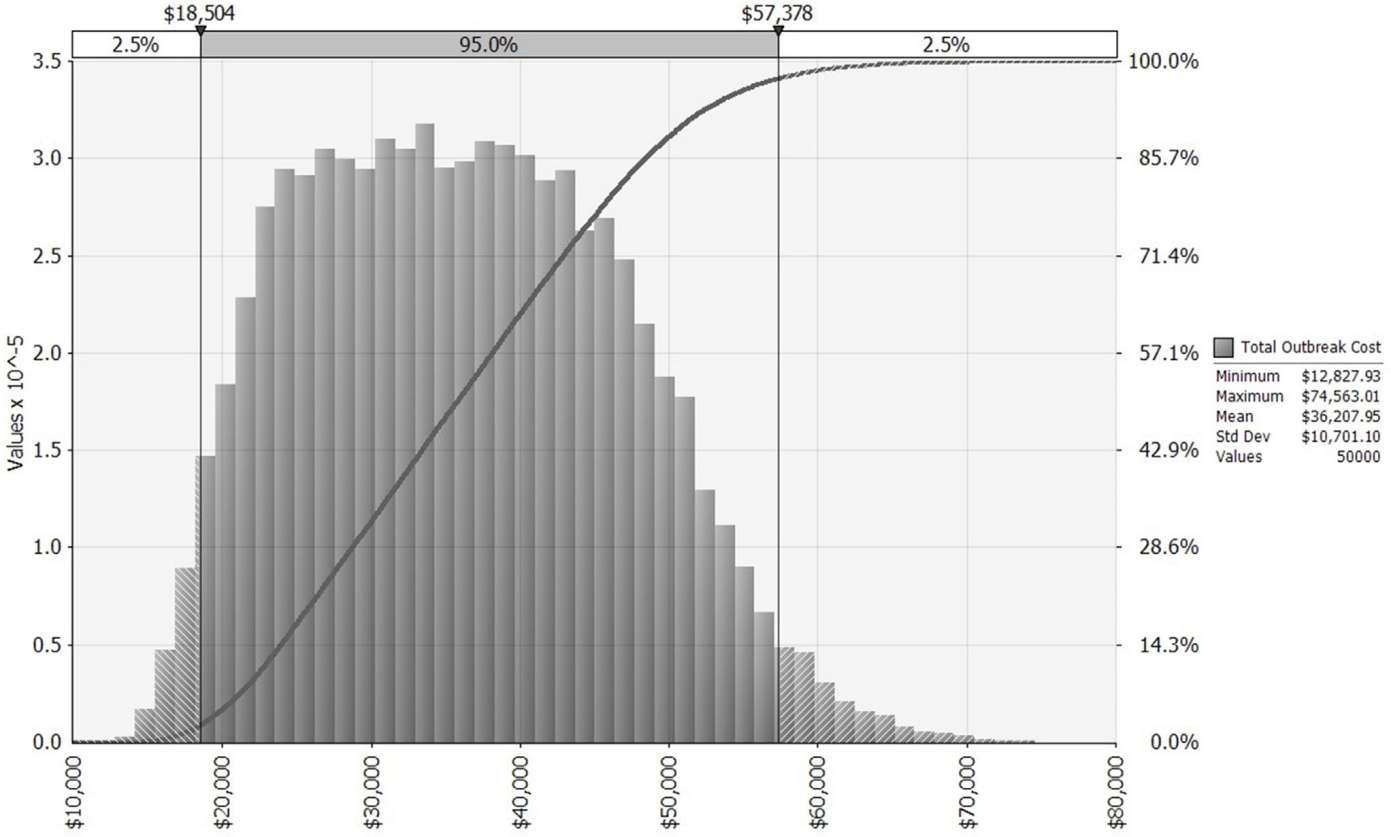
Distribution of bluetongue outbreak costs for a 640-ewe operation, allowing morbidity, mortality, and lamb price to vary across 50,000 iterations, based on historical distributions of these three random variables. *x*-Axis shows outbreak costs measured in US$2014. The left vertical axis and gray bars present a histogram for outbreak cost. The right vertical axis and dark gray curve present a cumulative probability distribution for outbreak cost [i.e., pr(Outbreak Cost) ≤ $X = Y%].

We first simulate a distribution for outbreak cost (Figure 2), which then serves as an intermediate input to the simulation of distributions for expected annual net benefit, one each for the KV and MLV vaccines. Each of these three distributions is influenced by the input distributions chosen for lamb price, morbidity rate, and mortality rate. We fit a log-logistic distribution to observed lamb prices (28) from the Fort Collins auction barn during November 1990 to November 2014 (adjusted to US$2014; see Figures S1 and S2 in Supplementary Material). Its parameters are set to the following values: γ = 0.58805, β = 128.31, α = 7.0474, minimum = 46.53, maximum = 229. Truncation at the minimum and maximum values allows the lamb price to fall one SD ($0.31/pound) below or above the historical price range.

We model the morbidity rate as a uniform distribution with a minimum = 0.06 and maximum = 0.36. Similarly, we model the mortality rate as a uniform distribution with a minimum = 0.046 and maximum = 0.20. Parameter values for the mortality and morbidity rates are based on observations from a previously naïve flock in the Big Horn Basin (for maximum values), and from a routinely exposed flock in Johnson County, Wyoming (for minimum values). More details of the rationale behind the distributions chosen are presented in Munsick [(24), p. 32–36]. Table S3 in Supplementary Material provides @Risk formulas for the three input distributions.

## Results and Discussion

### BT Outbreak Costs

Table 1 provides outbreak costs assuming mean values for morbidity rate (21%), mortality rate (12.3%), and lamb price ($1.30 per pound). Figure 2 depicts the distribution of simulated outbreak costs for the operation, accounting for variability in lamb price, morbidity, and mortality. Costs associated with a BT outbreak can be disaggregated into several categories.

**Table 1:** Bluetongue outbreak costs for a 640-ewe operation (US$2014).

**Supportive care costs**		**Death loss**	
Creep feed costs		Lamb death loss	9,677
Lambs (lived + died)	1,235	Ewe death loss	15,161
Ewes (lived + died)	1,783	Ram death loss	1,275
Rams (lived + died)	54	*Total death loss*	$26,113
Pharmaceutical costs		**Other costs and losses**	
Permethrin (insecticide spray)	38	Lamb weight loss	684
Nuflor (pneumonia prevention)	2,832	Veterinarian cost	0
Dexamethasone (inflammation)	28	Labor cost	3,439
*Total supportive care costs*	$5,970	*Total other*	$4,123
	
		Total outbreak cost	$36,206
		% of Baseline profit	35%

*Reported costs are calculated using the mean morbidity rate (21%), mean mortality rate (12.3%), and mean lamb price ($1.30/pound)*.

Total death loss is the combined loss of ewes, rams, and lambs due strictly to a BT outbreak. Although ram death loss is static, ewe death loss is a function of the variable lamb price and is, therefore, a distribution. After all, the death of a ewe triggers retention of an extra replacement lamb, which would have otherwise been sold at the variable lamb price. Lamb death loss is also a function of the variable lamb price, as well as morbidity and mortality rates.

Supportive care costs include tubing infected animals with a creep feed/water mixture and administering necessary pharmaceuticals as needed. Creep feed costs are driven by the variable morbidity and mortality rates. Pharmaceutical costs include direct application of permethrin insecticide spray, administration of Nuflor for all clinically infected cases, and administration of dexamethasone as needed. Additional costs include weight loss on surviving infected lambs, labor, and veterinarian costs (only those paid by the producer).

### BT Vaccination Costs

The cost of vaccination includes both the cost of purchasing vaccine and the labor to administer it. Cost of hired labor is assumed to be $10.64/h (31) adjusted by a producer price index of 1.091. Purchase price of the vaccine is estimated at $0.32/dose for the MLV vaccine and $1.20/dose for the KV vaccine. Table 2 shows the vaccine and labor costs for vaccinating the entire flock, using the MLV versus KV vaccine. Costs reported for the KV vaccine include both the first dose and the required follow-up dose.

**Table 2:** Cost of annual bluetongue vaccination for a 640-breeding-ewe operation (entire flock at time of vaccination is 1,423 sheep) using a modified-live virus (MLV) versus killed virus (KV) vaccine (US$2014).

	MLV	KV
# Doses required per sheep	1	2
Total vaccine cost	455	3,416
Total labor cost	42	84
Total cost per year	$497	$3,500
Average cost per sheep	$0.35	$2.46

### BT Vaccination Benefits

The benefit of vaccinating sheep against BT is a reduction in the proportion of the flock affected when the virus strikes. In a 640-ewe flock that has been vaccinated, the average number of morbidities and mortalities is reduced from 285 and 167 (without vaccination), respectively, to just 46 and 27 (with vaccination). This reduces the cost of an outbreak from an average of $36,207 (without vaccination) to $5,243 (with vaccination). Thus, on average, the benefit of vaccinating is $30,964 per outbreak. This benefit ranges, however, from as little as $11,325 to as much as $63,183, depending on the outbreak’s severity and the lamb price. Because the benefit of vaccinating depends so heavily on outbreak cost, their distributions are shaped similarly (compare Figure 2 to Figure S3 in Supplementary Material).

**Figure 3:**
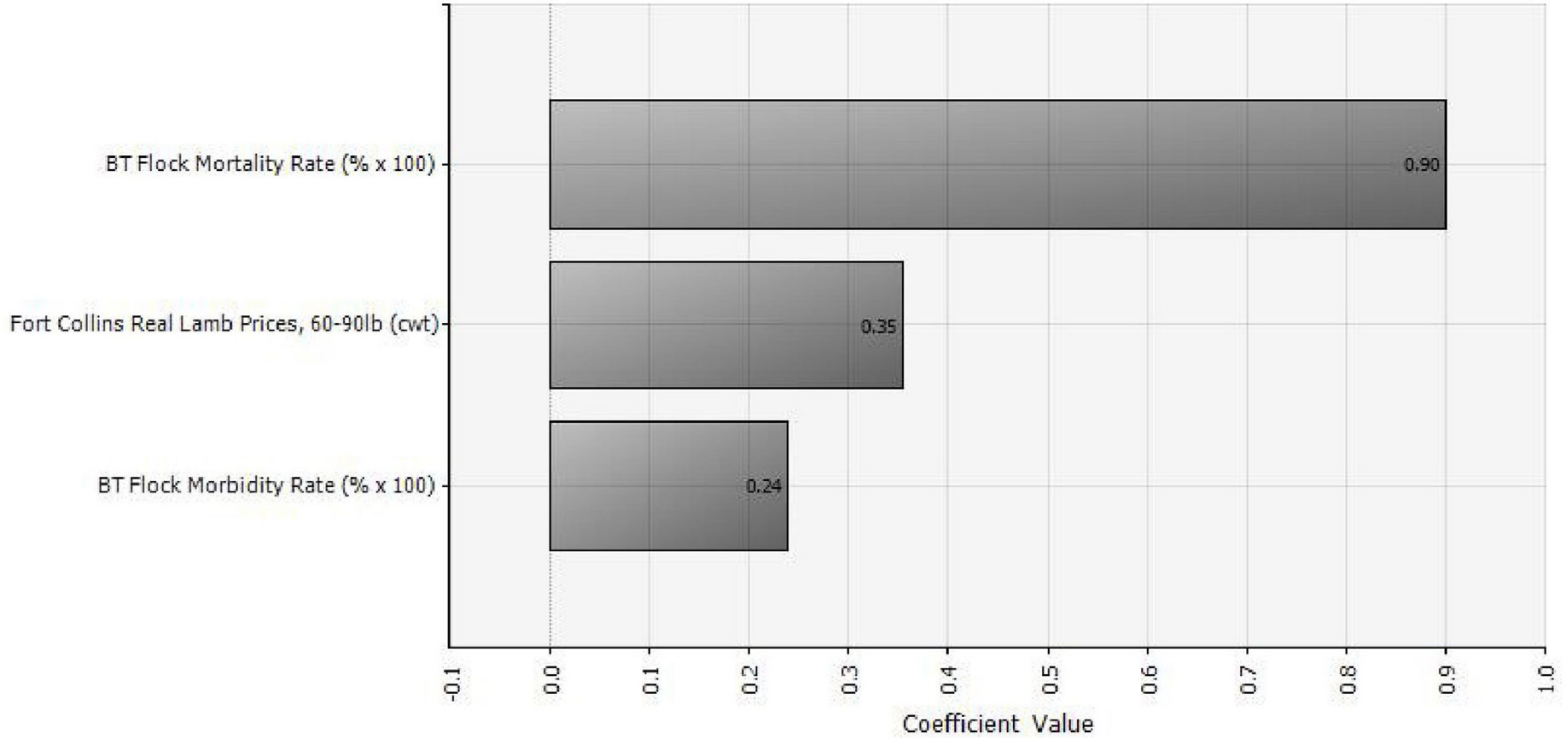
Relative influence of three different random variables (lamb prices, morbidity, and mortality) on the benefit of vaccinating against Bluetongue disease (with either modified-live virus or killed virus), assuming an annual vaccination strategy.

Of the three random variables in our analysis, BT mortality rate has the greatest influence on outbreak cost and hence vaccination benefit. Lamb price has the second greatest influence, followed by BT morbidity rate. Figure 3 shows a tornado graph (using the “regression coefficients” option in @Risk) of relative influence of the three random variables on vaccination benefit. Regression coefficients indicate the amount of change that will occur in a dependent variable due to a change in an independent variable. For example, increasing the lamb price by 1 unit will result in a 0.35 unit increase in the benefit of vaccination.

The total benefit of administering a BT vaccine, over a producer’s career, depends on how often their flock is exposed to the virus. Ideally, a producer would only have to vaccinate in years when the virus is known to be a threat. Unfortunately, society’s ability to predict an outbreak is currently limited, so producers must decide ahead of the risk season each year whether to vaccinate. Before making the vaccination decision, producers should compare the annual cost of vaccinating against its expected annual benefit, to determine expected annual net benefit.

### BT Vaccination Net Benefits

**Figure 4:**
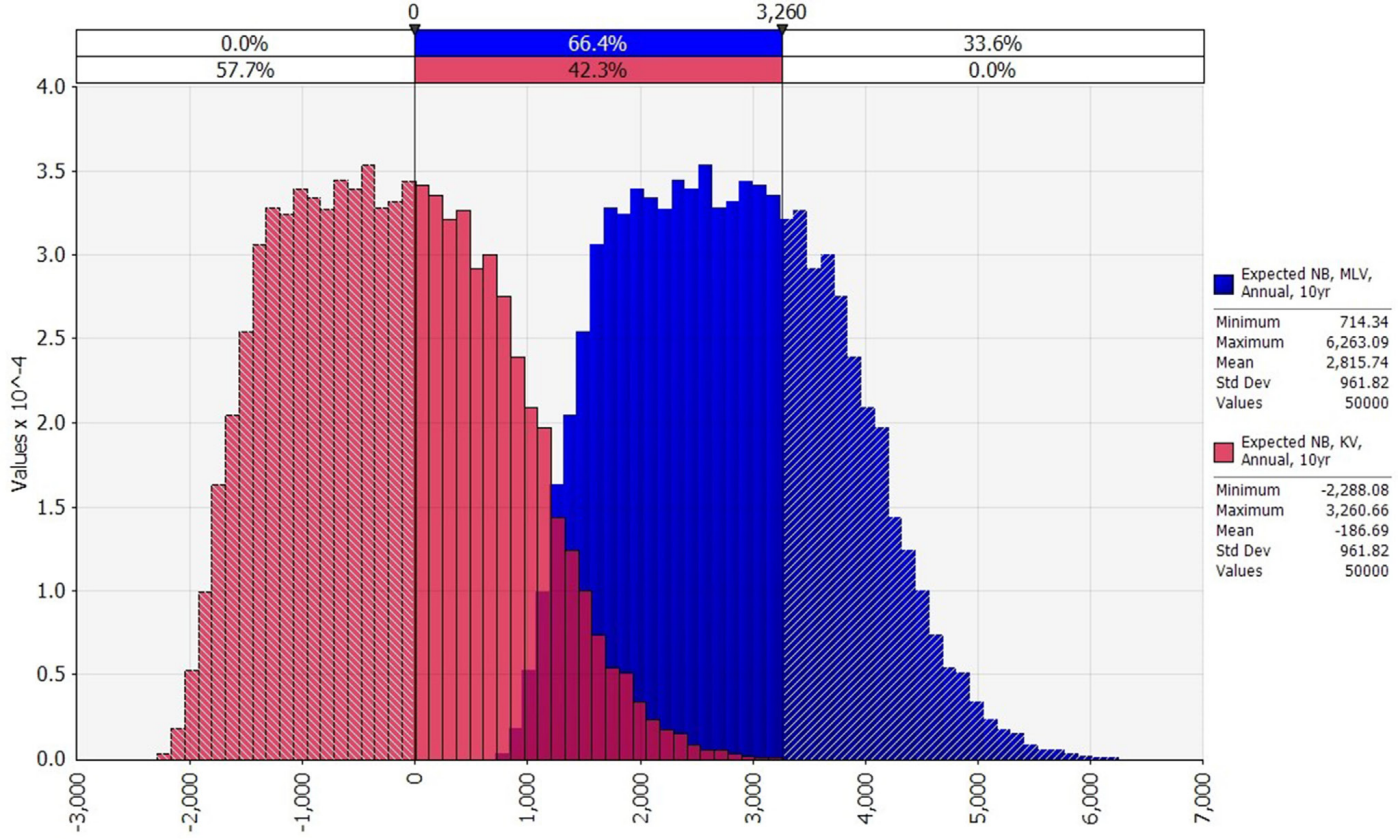
Comparison of the distributions of expected annual net benefit for modified-live virus (MLV) and killed virus (KV) (US$2014), assuming an annual vaccination strategy, and 10 years between outbreaks. Variability in any one of the vaccine’s expected annual net benefit is due to variability in lamb price, Bluetongue (BT) morbidity rate, and BT mortality rate. MLV’s net benefit exceeds the maximum net benefit of KV in 33.6% of the 50,000 iterations.

**Figure 5:**
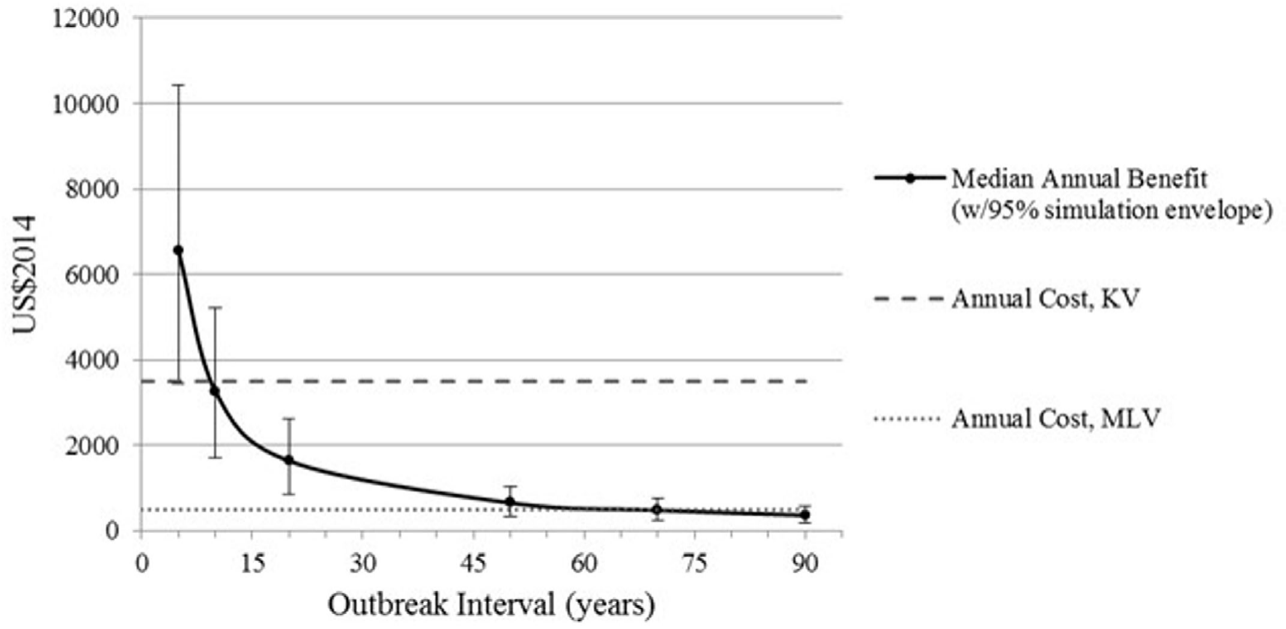
Median expected annual benefit for both modified-live virus (MLV) and killed virus (KV) vaccines, with bars reflecting the 95% simulation envelope (i.e., 2.5th and 97.5th percentile). Annual cost of MLV and KV vaccines. Intersection of a vaccine’s cost curve with the median benefit curve indicates the outbreak interval at which that vaccine’s median annual benefit no longer exceeds its annual cost. All benefits and costs are reported in US$2014.

To calculate expected annual net benefit of vaccination, we must assume how frequently the virus will strike. Table 3 reports the mean value of expected annual net benefit for outbreak intervals of 5, 10, 20, and 50 years. Expected annual net benefit of vaccination tends to be higher for MLV than for the KV vaccine (Table 3; Figure 4). This is because the MLV vaccine is cheaper to purchase and requires only one dose per year, whereas the KV vaccine has a higher assumed purchase price and requires a follow-up dose. The higher expected annual net benefit for MLV than for KV holds true across all outbreak intervals (Table 3). More specifically, MLV yields positive expected annual net benefit (based on its median) up to 69 years between outbreaks (Figure 5). KV vaccine, in contrast, yields positive expected annual net benefit up to just 9 years between outbreaks (Figure 5).

**Table 3:** Mean value of expected annual net benefit (US$2014) for two different vaccine types [modified-live virus (MLV), killed virus (KV)], administered annually, under different outbreak intervals (5, 10, 20, and 50 years).

	Outbreak interval			
	5 years	10 years	20 years	50 years
MLV	$6,129	$2,816	$1,159	$165
KV	$3,126	−$187	−$1,843	−$2,837

Figure 5 also reveals that, as outbreak interval lengthens, expected annual benefit decreases. This is because benefit is assumed to occur just once during a given interval. Thus, as outbreak interval lengthens, the annualized benefit of vaccination gets smaller and smaller, whereas the annual vaccination cost remains the same. This causes the expected annual net benefit to decrease as outbreak interval lengthens (Table 3).

Turning to variability, the distributions of expected annual net benefit for MLV versus KV are shown in Figure 4, for a 10-year outbreak interval. The shape of these distributions is similar to those for 5, 20, and 50-year outbreak intervals (not shown). Note that the two distributions in Figure 4 are identically shaped and differ only in their mean values. This is because they both derive from the same outbreak cost distribution (Figure 2), which is based on a log-logistic lamb price distribution (Figure S1 in Supplementary Material) and uniform morbidity and mortality distributions (Table S3 in Supplementary Material). The identical distribution of expected annual net benefit for MLV versus KV (Figure 4) also reflects our assumption that both vaccines are equally effective. Given their many similarities, the two distributions differ only because the MLV vaccine is assumed to be $3,003 cheaper than KV (precisely the difference between the two distributions’ means). The purpose of Figure 4 is to illustrate the dramatic implication this cost difference has on the probability of MLV versus KV generating positive expected annual net benefit. For a 10-year outbreak interval, MLV has a 100% chance of generating positive expected annual net benefit, whereas KV has only a 42% chance. This cost-driven reduction in the probability of breaking even, on average, will reduce producers’ willingness to adopt KV as compared to MLV.

Table 4 reports, for various outbreak intervals, the percent of simulation iterations in which vaccination enjoys positive expected annual net benefit. The KV vaccine’s probability of yielding positive expected net benefit falls sharply from 97 to 42% as outbreak interval lengthens from 5 to 10 years. And for producers facing a 20-year or longer outbreak interval, there is zero probability that a KV vaccine will generate positive expected net benefit. For the MLV vaccine, in contrast, there is a 100% chance of positive expected net benefit for outbreak intervals of 5, 10, and 20 years. Only at an outbreak interval of 50 years does this probability fall to 77%. This reflects, again, the MLV vaccine’s lower assumed cost, and highlights its important economic implications.

**Table 4:** Percentage of iterations in which vaccination with modified-live virus (MLV) versus killed virus (KV) has positive expected annual net benefit, for various outbreak intervals, on a 640-ewe operation.

	Vaccination strategy	
Outbreak interval (years)	MLV (%)	KV (%)
5	100	97
10	100	42
20	100	0
50	77	0

### Epizootic versus Endemic Outbreaks

A sheep operation’s location and history of disease play an important role in the expected net benefit of vaccinating. Producers in high-frequency, low-virulence areas, who face regular but mild outbreaks, may face a different risk than producers in low-frequency areas with no recent history of the disease and thus naïve sheep flocks and severe outbreaks.

Table 5 reports two iterations from the @Risk simulation to put expected annual net benefit of vaccination (using an MLV vaccine) into a geographic context. We have hand-selected two iterations that share the same historical mean lamb price, $130.36 (25), but exhibit different outbreak characteristics. One iteration represents conditions typical of the Big Horn Basin, where sheep flocks have experienced outbreaks relatively infrequently (e.g., roughly every 10 years) and relatively severely (e.g., 22% morbidity, 20% mortality). The other iteration represents conditions typical of eastern Wyoming, where flocks have experienced outbreaks more frequently (e.g., roughly every 5 years) yet less severely (e.g., 6% morbidity, 7% mortality). The Big Horn Basin iteration is chosen from among the 50,000 MLV iterations underlying Figure 4 (i.e., a 10-year outbreak interval). The eastern Wyoming iteration is chosen from among 50,000 MLV iterations underlying a similar (unpublished) figure for a 5-year outbreak interval.

**Table 5:** Two hypothetical outbreaks in Wyoming, depicting two different iterations from a single simulation—one akin to eastern WY (more frequent but less severe outbreaks) and one akin to the Big Horn Basin (less frequent but more severe outbreaks)—using annual vaccination with a MLV vaccine on a 640-ewe operation.

	Eastern WY	Big Horn Basin
Outbreak interval (years)	5	10
Morbidity rate	0.06	0.22
Mortality rate	0.07	0.20
Lamb price (per hundredweight)	$130.36	$130.36
Annual net benefit	$3,253	$4,329

Vaccination is less beneficial for producers located in a “lower” risk (i.e., more frequent thus less severe outbreaks) area compared to those in a “higher” risk (i.e., less frequent thus more severe outbreaks) area (Table 5). Ironically, producers that experience outbreaks less frequently may also be less likely to administer the vaccine (32). For producers in areas with shorter outbreak intervals and lower risk of high-severity outbreaks (e.g., eastern Wyoming), lamb prices may be a significant factor in their decision to vaccinate or not. Vaccination might not be profitable in years with unusually low lamb prices. Increased BT forecasting would give producers in low-risk areas the ability to vaccinate only in years when lamb prices are high enough to make vaccination profitable, thereby decreasing vaccination costs. This is a promising area for future research.

### Implications for Vaccine Approval in Wyoming

We have modeled and compared the costs and benefits for both MLV and KV vaccines. Recall, however, that the MLV vaccine requires a rigorous authorization process before legal distribution and use can occur in Wyoming. To date, no company has requested to make an MLV vaccine commercially available for use in Wyoming. It may be more feasible for Wyoming producers to obtain approval for a custom-made KV vaccine because it poses less risk to sheep reproduction and less risk of escape. However, based on our estimated purchase price of $1.20 per dose, a KV vaccine would be seven times more expensive to manufacture and administer than an MLV vaccine (Table 2). This is sufficiently expensive that a producer who faces an outbreak interval longer than 5 years is unlikely to adopt it because of its low probability of generating positive expected annual net benefit. An MLV vaccine, in contrast, has a high probability of generating positive expected annual net benefit for producers who face even a 50-year outbreak interval.

This suggests further research is needed to quantify the value of vaccine safety and determine whether it is high enough to justify the current ban on MLV vaccines. The MLV vaccine’s risk of escape would need to be sufficiently costly to make the MLV vaccine’s cost equal to or greater than the KV vaccine’s cost. If, however, MLV were shown to be less expensive than KV, even after accounting for the external costs of potential escape, then rules banning MLV vaccines may be economically inefficient.

The risk of escape is real, not simply hypothetical. A recent outbreak of BTV-3 in India, for example, was traced back to Western virus strains, and is believed to have been initiated by reassortment of the virus through MLV vaccination (33). External costs of MLV vaccination may be difficult to quantify, but further research is needed so animal health officials can make economically informed decisions regarding the most efficient type of vaccination against BT disease.

### Limitations

The outbreak costs estimated in this paper are large enough to pose an economic threat to producers, yet do not fully reflect the damage that a severe outbreak can inflict on an individual operation. Intangible costs such as negative impacts on personal health, family dynamics, and community relationships are also important. An outbreak of this magnitude may have severe and long-lasting effects on an individual or family and their operation (Personal communication with Ranch A operator in 2015). We do not attempt to place an economic value on these intangible impacts and have, therefore, underestimated the cost of a BT outbreak and, subsequently, the expected annual net benefit of vaccinating against it.

In addition to expected annual net benefit—the focus of our analysis—risk preference and other behavioral tendencies are likely to influence an individual producers’ vaccination choice. Risk preferences range from risk-loving to risk-averse, with risk-neutral falling between the two. We have analyzed BT vaccination from the perspective of a risk-neutral producer, i.e., one who cares only about maximizing the expected value of a decision, without concern for potential variability in the outcome of that decision. In contrast, we would expect a risk-averse producer to be more likely to vaccinate their flock against BT. Given the robustness of our results for the MLV vaccine—indicating a positive expected annual net benefit under a wide range of possible conditions—inclusion of risk aversion would reinforce our findings that an MLV vaccine against BT is likely to make economic sense for many sheep producers in at-risk areas of the Intermountain West. Inclusion of risk aversion would also increase the economic attractiveness of a KV vaccine for producers in areas with outbreak intervals longer than 5 years. We do not attempt to determine, however, how large an effect this would have to be to overcome the KV vaccine’s predominantly negative expected annual net benefit for longer outbreak intervals.

## Author Contributions

TM: acquisition of the data, analysis and interpretation of data for the work, drafting the work and revising it critically for important intellectual content, and final approval of the version to be published. DP: conception and design of the work, interpretation of the data, revising the manuscript critically for important intellectual content, and final approval of the version to be published. JR: data analysis and interpretation, revising the manuscript critically for important intellectual content, and final approval of the version to be published. RJ and MJ: data acquisition, revising the data, and final approval of the version to be published. MM: conception of the work, data acquisition, revising the manuscript for critically important intellectual content, and final approval of the version to be published. All authors agreed to be accountable for all aspects of the work in ensuring that questions related to the accuracy or integrity of any part of the work are appropriately investigated and resolved.

## Conflict of Interest Statement

TM, DP, JR, RJ, and MM did not receive payment or services from any third party, besides the funding agencies named earlier; they have no financial relationships with entities that could be perceived to influence the submitted work. No financial incentives were provided to sheep producers or veterinarians providing information for the study.
