# Supplementary material for: Expected Net Benefit of Vaccinating Rangeland Sheep against Bluetongue Virus Using a Modified-Live versus Killed Virus Vaccine
Source: Front Vet Sci. 2017 Oct 11;4:166. doi: 10.3389/fvets.2017.00166 (PMC5641540; doi:10.3389/fvets.2017.00166)
Supplement: Supplementary file 1 [file table_1.pdf]

SUPPLEMENTARY MATERIAL

Table S.1. Variable input and output prices assumed in the sheep enterprise budgets, along with the sources used to update them to US$2014. Any prices not explicitly listed here were obtained from Gardiner (2011) and updated using PPIs from Wyoming Ag Statistics as described in Munsick (2016, p. 76).

**Variable Inputs**	**Unit**	**Price/Unit**	**Source**
Alfalfa/Grass Mix Hay	ton	$188.00	USDA NASS (2014)
2nd Cut Alfalfa Hay (high TDN, CP)	ton	$193.00	USDA NASS (2014)
Cake (14% CP)	ton	$140.00	Camino (2015)
Creep Feed	lb	$0.34	Murdoch's Farm and Ranch Supply (2015)
Nuflor	cc	$0.62	Cherni (2015)
Dexamethasone	cc	$0.02	Cherni (2015)
Permethrin 10% Concentrate	pint	$18.99	Murdoch's Farm and Ranch Supply (2015)
State Grazing Allotment	AUM	$5.54	State of Wyoming Office of Lands and Investments (2015)
Federal Grazing Allotment	AUM	$1.69	U.S. Department of Interior (2015)
Hired Labor	hr	$10.64	U.S. Department of Labor (2009)* PPI_{2014} for wages of 1.091
Replacement Rams	ram	$1,097	Wyoming Woolgrowers Association (2015)
**Outputs**	**Unit**	**Price/Unit**	**Source**
Market Lambs	cwt	Distribution (avg: $1.30/lb)	LMIC (2015) for Feeder Lambs (60-90 lb), Ft. Collins, 1990-2014
Cull Ewes	cwt	$37.00	LMIC (2015) for Cull Ewes, Ft. Collins, on 8/23/14
Wool	lb	$2.13	USDA NASS (2014)

Table S.2. Summary of the annual budget for a 640 breeding-ewe flock using US$2014 prices.

**Total Receipts ($)**	95,347
**Total Costs ($)**	115,386
**Returns To Land, Labor & Management ($)**	(20,039)
**Net Income ($)**	(102,972)
**Flock Dynamics:**	
Number of Breeding Ewes	640
Number of Breeding Rams	20
Number of Purchased Repl. Ewes	23
Number of Purchased Repl. Rams	5
Ewe Replacement Rate (% x 100)	0.15
Ewe Cull Rate (% x 100)	0.15
Ram Cull Rate (% x 100)	0.20
Proportion of Lambs born in *1st Estrus*	0.60
Proportion of Lambs born in 2nd *Estrus*	0.24
Proportion of Lambs born in *3rd Estrus*	0.16
**Receipts ($):**	
Lamb Receipts ($1.98 per pound)	75,119
Cull Ewes Receipts	5,869
Cull Rams Receipts	322
Wool Sales Receipts ($2.13 per pound)	14,037
**Total Receipts ($)**	95,347
**Variable Costs ($):**	
Alfalfa Hay ($188 per ton)	36,593
Grain	11,693
Salt / Minerals	2,094
Federal Grazing Allot.	2,165
State Grazing Allot.	5,137
Grazing Association Fees	640
Replacement Rams	5,486
Replacement Ewes	1,555
Shearing (ewes)	176
Shearing (rams)	109

**Variable Operating Costs (cont'd):**	
Sheep Identification	634
Veterinary / Medicine	3,399
Hauling / Trucking	6,253
Predator Control	2,830
Hired Labor	9,485
Seasonal Herding Labor	3,682
Camp Supplies	3,282
Horse Care	1,307
Dog Food	2,038
Tractor	8,456
Pickup	2,138
Four Wheeler	122
Hay Wagon	48
Posthole Digger	8
Machinery Repair Costs	960
Building Repair & Improvement	91
Fencing	5,005
**Total Variable Costs ($)**	115,386
**Fixed Costs ($):**	
Equipment Depreciation	12,187
Building Depreciation	1,890
Horse Depreciation	1,467
Herd/Guard Dog Depreciation	60
Utilities	4,282
Building Insurance	8,066
Vehicle Insurance	1,641
Farm Liability Insurance	850
Property Tax	22,680
Taxes and Registration	8,496
Interest on Long Term Liabilities	16,698
Interest on Operating Capital	2,965
Accountant & Legal Fees	1,653
**Total Fixed Costs ($)**	82,934

Table S.3. @Risk formulas used to represent the distribution of lamb price, morbidity rate, and mortality rate.

**Random Variable**	**Source**	**@Risk Formula**	**Mean**	**Median**
US$2014 Price for Feeder Lambs (60-90 lb), Ft. Collins, 1990-2014	LMIC (2015)	RiskLoglogistic(0.58805, 128.31,7.0474,RiskTruncate(46.53,229))	$129.91	$127.16
Morbidity Rate	Munsick (2016, pp32-36)	RiskUniform(0.060,0.36)	0.210	0.210
Mortality Rate	Munsick (2016, pp32-36)	RiskUniform(0.046,0.20)	0.123	0.123

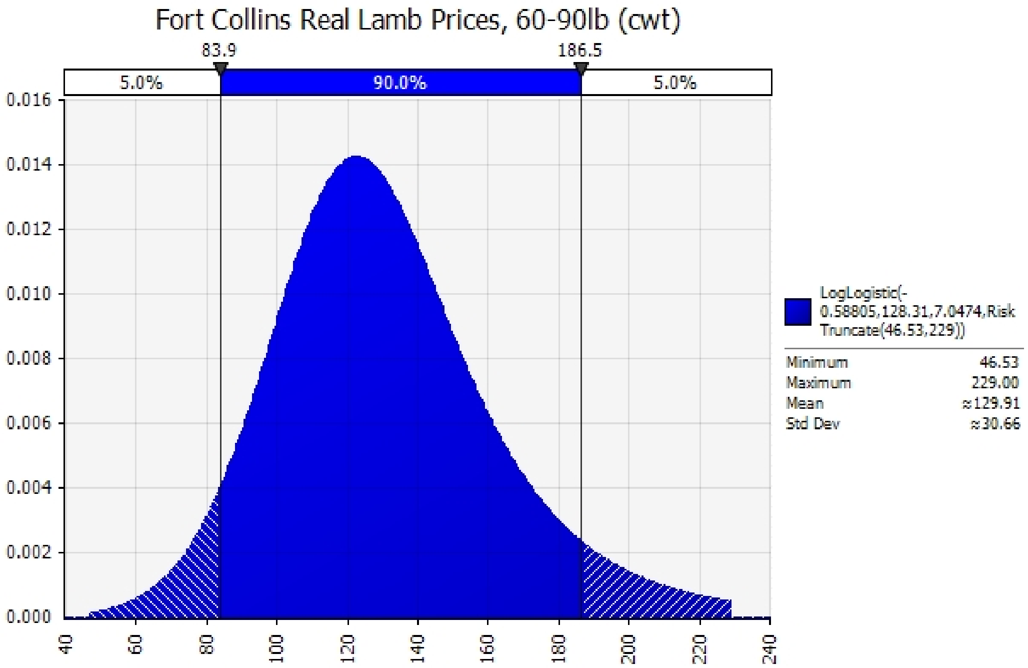

Figure S.1. Log-logistic distribution used to represent historical feeder lamb (60-90 lb.) prices from the Fort Collins auction, November 1990 to November 2014, adjusted to US$2014. Source: Livestock Marketing Information Center; retrieved from http://www.lmic.info/page/key-graphs?q=user.

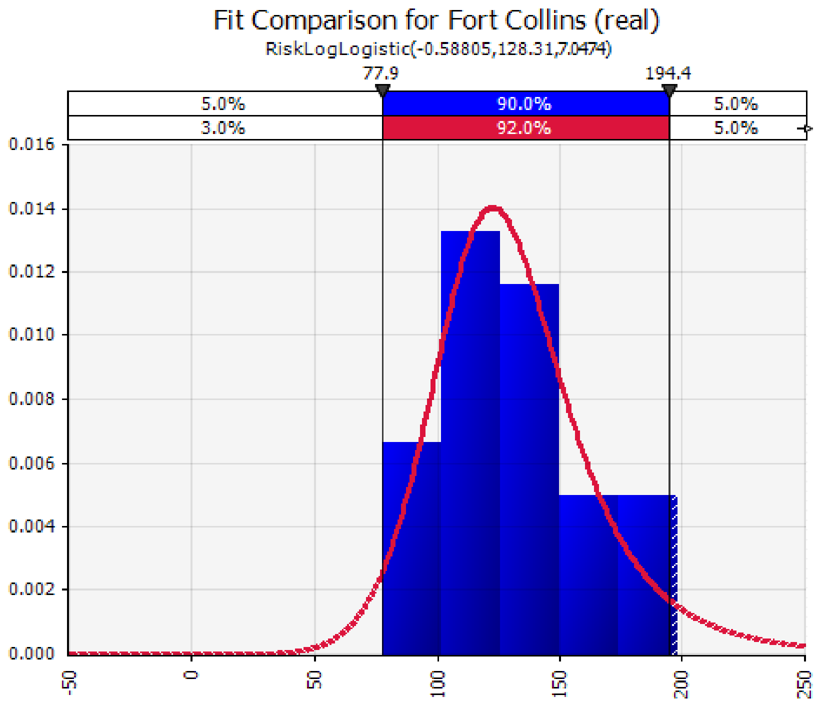

Statistics

	Input	LogLogistic
Minimum	77.56	-0.588
Maximum	198.06	+∞
Mean	131.42	132.07
Mode	≈115.36	122.62
Median	128.77	127.72
Std Dev	32.97	35.59
Skewness	0.4355	1.4445
Kurtosis	2.7030	10.2317
Left X	77.9	77.9
Left P	5.0%	3.0%
Right X	194.4	194.4
Right P	95.0%	95.0%
Dif. X	116.53	116.53
Dif. P	90.0%	92.0%
1%	77.56	66.26
5%	77.86	83.90
10%	91.09	93.35
15%	96.06	99.73
20%	105.55	104.81
25%	114.90	109.20
30%	115.51	113.19
35%	115.66	116.93
40%	116.49	120.55
45%	123.44	124.12

Figure S.2. Histogram (in blue) of historical feeder lamb (60-90 lb.) prices from the Fort Collins auction, November 1990 to November 2014, adjusted to US$2014. Log-logistic distribution (in red), prior to truncation, as used to represent historical feeder lamb prices. Source: Livestock Marketing Information Center; retrieved from http://www.lmic.info/page/key-graphs?q=user.

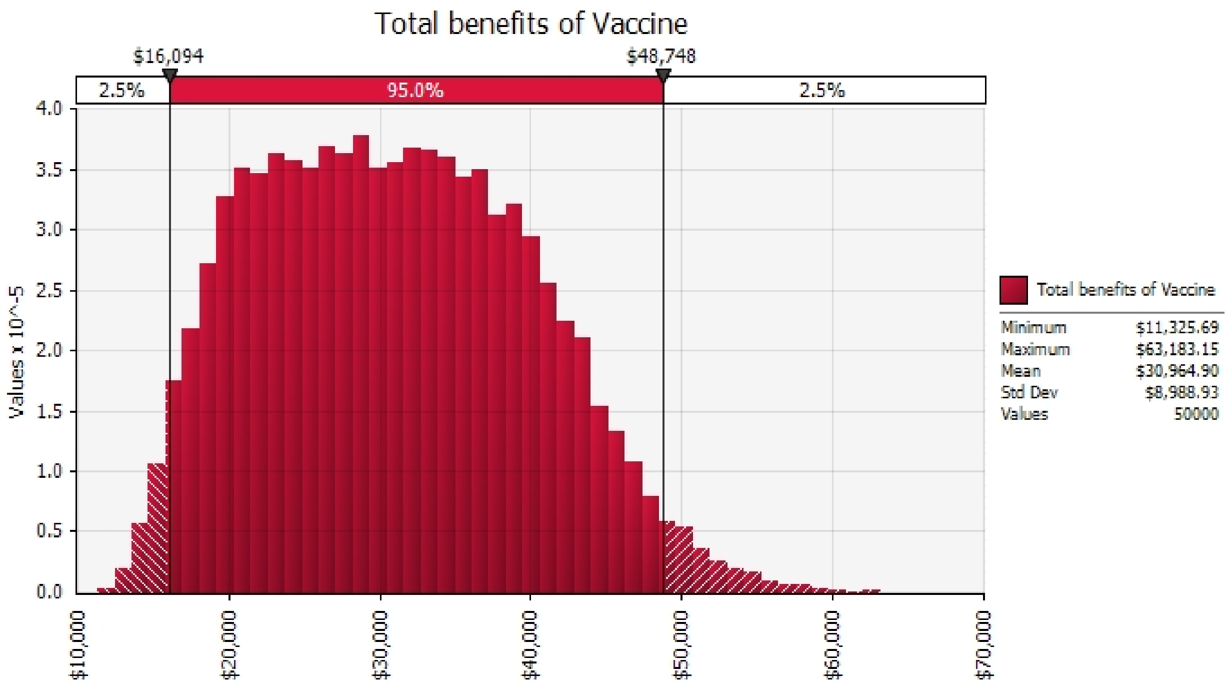

Figure S.3. Distribution of bluetongue vaccination benefits for a 640-ewe operation, allowing morbidity, mortality, and lamb price to vary across 50,000 iterations according to their historical distributions. We assume a vaccine is 84% effective; therefore, the benefit of vaccination is roughly equal to 84% of outbreak cost. Actual calculations of benefit are more complex than directly scaling outbreak cost by 84%, as we describe in the main text. Nonetheless, these calculations do generate a benefit distribution similar in shape to the outbreak cost distribution (see figure 2 in main text).

REFERENCES

Camino P. Personal communication by phone with Peter Camino, former President of Wyoming Woolgrowers Association (September 10, 2015).

Cherni M. Personal communication by phone with Matt Cherni, DVM, practicing large animal veterinarian (August 15, 2015).

Gardiner LK. *Economics of Ram Mating Behavior [Master's Thesis]*. Laramie, WY: University of Wyoming (2011).

Livestock Marketing Information Center (LMIC). *Data from: Cull Ewes, Fort Collins Auction, August 23, 2014*. (2015).

Munsick TR. *Economics of Vaccinating Rangeland Sheep Flocks against Bluetongue Virus [Master's Thesis]*. Laramie, WY: University of Wyoming (2016).

Murdoch's Farm and Ranch Supply. Personal communication by phone interview with Murdoch's Farm and Ranch Supply salesman (June 4, 2015).

State of Wyoming, Office of Lands and Investments. *Office of State lands and Investments Annual Report 2012*. Cheyenne, WY (June 25, 2015). Available from: http://wwwwsl.state.wy.us/slpub/reports/State%20Lands%20Annual%20Report(RL).pdf

United States Department of Agriculture, National Agricultural Statistics Service. *Wyoming Agricultural Statistics*. Cheyenne, WY: Wyoming Field Office (2014). 95 p.

United States Department of the Interior, Bureau of Land Management. *BLM and Forest Service Announce 2015 Grazing Fee* (January 30, 2015). Available from: https://www.blm.gov/press-release/blm-and-forest-service-announce-2015-grazing-fee-0

United States Department of Labor, Bureau of Labor Statistics. *Occupational Employment and Wages–May 2009: 45-2093 Farmworkers, Farm and Ranch Animals (Wyoming Median Hourly Wage)*. Washington, DC (2009). Available from: https://www.bls.gov/oes/special.requests/oesm09st.zip

Wyoming Woolgrowers Association. *2014 Wyoming State Ram Sale, Average Rambouillet*. Glenrock, WY (2015). Available from: http://wyowool.com/Ram%20Sale/2014%20Sale/2014SaleResults.pdf
